# Improving midwifery educators’ capacity to teach emergency obstetrics and newborn care in Kenya universities: a pre-post study

**DOI:** 10.1186/s12909-022-03827-4

**Published:** 2022-10-31

**Authors:** Duncan N Shikuku, Joyce Jebet, Peter Nandikove, Edna Tallam, Evans Ogoti, Lucy Nyaga, Hellen Mutsi, Issak Bashir, Dan Okoro, Sarah Bar Zeev, Charles Ameh

**Affiliations:** 1 Liverpool School of Tropical Medicine (Kenya), P.O. Box 24672-00100, Nairobi, Kenya; 2 Liverpool School of Tropical Medicine (UK), L3 5QA Liverpool, UK; 3 University of Nairobi, P. O. Box 19676-00100, Nairobi, Kenya; 4 Masinde Muliro University of Science and Technology, P.O. Box 190-50100, Kakamega, Kenya; 5 Nursing Council of Kenya, P.O. Box 20056-00200, Nairobi, Kenya; 6 Moi University, P.O. Box 4606 – 30100, Eldoret, Kenya; 7 Department of Family Health, Ministry of Health, P.O. Box 30016-00100, Nairobi, Kenya; 8 United Nations Population Fund Kenya, P.O. Box 30218-00100, Nairobi, Kenya; 9 Technical Division, United Nations Population Fund, 10158 New York, NY USA

**Keywords:** Pre-service, Midwifery educators, Emergency obstetrics and newborn care, Curriculum, Kenya

## Abstract

**Background:**

International Confederation of Midwives and World Health Organization recommend core competencies for midwifery educators for effective theory and practical teaching and practice. Deficient curricula and lack of skilled midwifery educators are important factors affecting the quality of graduates from midwifery programmes. The objective of the study was to assess the capacity of university midwifery educators to deliver the updated competency-based curriculum after the capacity strengthening workshop in Kenya.

**Methods:**

The study used a quasi-experimental (pre-post) design. A four-day training to strengthen the capacity of educators to deliver emergency obstetrics and newborn care (EmONC) within the updated curriculum was conducted for 30 midwifery educators from 27 universities in Kenya. Before-after training assessments in knowledge, two EmONC skills and self-perceived confidence in using different teaching methodologies to deliver the competency-based curricula were conducted. Wilcoxon signed-rank test was used to compare the before-after knowledge and skills mean scores. McNemar test was used to compare differences in the proportion of educators’ self-reported confidence in applying the different teaching pedagogies. P-values < 0.05 were considered statistically significant.

**Findings:**

Thirty educators (7 males and 23 females) participated, of whom only 11 (37%) had participated in a previous hands-on basic EmONC training – with 10 (91%) having had the training over two years beforehand. Performance mean scores increased significantly for knowledge (60.3% − 88. %), shoulder dystocia management (51.4 – 88.3%), newborn resuscitation (37.9 − 89.1%), and overall skill score (44.7 − 88.7%), p < 0.0001. The proportion of educators with confidence in using different stimulatory participatory teaching methods increased significantly for simulation (36.7 – 70%, p = 0.006), scenarios (53.3 – 80%, p = 0.039) and peer teaching and support (33.3 – 63.3%, p = 0.022). There was improvement in use of lecture method (80 – 90%, p = 0.289), small group discussions (73.3 – 86.7%, p = 0.344) and giving effective feedback (60 – 80%, p = 0.146), although this was not statistically significant.

**Conclusion:**

Training improved midwifery educators’ knowledge, skills and confidence to deliver the updated EmONC-enhanced curriculum. To ensure that midwifery educators maintain their competence, there is need for structured regular mentoring and continuous professional development. Besides, there is need to cascade the capacity strengthening to reach more midwifery educators for a competent midwifery workforce.

**Supplementary Information:**

The online version contains supplementary material available at 10.1186/s12909-022-03827-4.

## Introduction

Quality midwifery education underpins the provision of quality midwifery care and is vital for the health and well-being of women, infants, and families. The third State of the World’s Midwifery Report (SoWMY 2021) identified that midwives fully educated to international standards and supported by interdisciplinary teams working within an enabling environment can deliver about 90% of essential sexual, reproductive, maternal, newborn and adolescent health (SRMNAH) interventions across the lifespan [1]. Despite this fact, midwives account for less than 10% of the global SRMNAH workforce worsened further by a global shortage of 900,000 midwives [1]. To make a significant impact on health outcomes and experiences in the health care system, midwives must be supported by an enabling environment and relevant regulations. The enabling environment includes drugs, supplies, appropriate policies, and a functional referral system [2, 3].

Midwives play a vital role in reducing maternal and perinatal mortality. Attaining universal coverage of midwifery delivered interventions – including skilled attendance at birth, provision of emergency obstetrics and newborn care (EmONC) and family planning by 2035 could prevent 67% of maternal deaths, 64% of newborn deaths and 65% of stillbirths – which translates to 4.5 million lives saved annually [4]. Despite the known benefits associated with midwifery education and training, it remains grossly under-invested with variation in the quality, content and duration of content between and within countries [1].

The lack of skilled midwifery educators is an important factor affecting the quality of graduates from midwifery programmes and evidence suggests that midwifery educators are more confident with theoretical classroom teaching than clinical teaching [5]. Other barriers to the delivery of quality midwifery education and training in low- and middle-income countries (LMICs) include lack of teaching resources, inconsistencies in the content and duration of midwifery curriculum, lack of access to clinical sites and connection with clinical practice and use of didactic teaching methods in traditional classroom settings despite advances in teaching methods [5–8]. These barriers faced by midwifery educators in their delivery of midwifery education thus result in their students graduating with outdated practices or lacking in expected clinical competencies [7].

The Kenya Health Policy 2014–2030 [9] and Kenya Health Sector Strategic Plan 2018–2023 [10] identified increasing access to equitable quality of care and services including emergency care and training of skilled and competent health workers as key national priority areas for achieving universal health coverage (UHC). Improving quality of midwifery education and training has been identified as a national priority to help reduce high burden of maternal and newborn mortality in Kenya. Key interventions highlighted in the Kenya Health Sector Strategic Plan 2018–2023 to achieve this include review of curricula to address emerging issues including UHC, strengthen the quality of training of pre-service faculty and clinical instructors on pedagogy skills and capacity building for provision of competency-based EmONC [10].

Midwifery education in Kenya is offered predominantly through the integrated nursing and midwifery programs in mid-level training colleges and universities at diploma and degree (bachelor, masters and doctoral) level. The predominant integrated nursing and midwifery training programs are Kenya Registered Community Health Nursing at diploma level and the Bachelor of Science in Nursing (BScN) at bachelor’s degree level. The other basic midwifery training programs offered include Kenya Registered Nursing and Midwifery and Kenya Registered Midwifery at diploma level and Bachelor of Science in Midwifery at degree level, the latter two being stand-along midwifery streams.

To improve the teaching competencies of midwifery educators and the quality of pre-service education and training in Kenya, the Nursing Council of Kenya (NCK) in collaboration with the Liverpool School of Tropical Medicine (LSTM) and multiple stakeholders in 2021 reviewed and updated the Bachelor of Science in Nursing curricula (direct-entry and upgrading) delivered in all the degree nurse/midwives training universities in Kenya. The review integrated the competency-based EmONC, defined as the collective minimum set of medical interventions (or bundle of care) required to prevent or manage the main obstetric complications (haemorrhage, pre-eclampsia or eclampsia, sepsis, complications of obstructed labour or abortion) and newborn complications (birth asphyxia) [11]. This competency-based EmONC was identified as a national priority need area that was deficient in the training of midwives as skilled health personnel in Kenya. This review aimed to contribute to the achievement of global and national UHC agenda and aligned to the World Health Organisation (WHO) and the International Confederation of Midwives (ICM)’s 2018 updated definition and competencies of the midwives as skilled health personnel with capacity to perform, as individuals or as part of a team, all signal functions of emergency obstetrics and newborn care [12, 13]. As recommended by ICM, midwifery curriculum should include both theory and practise essentials with a minimum of 40% theory and a minimum of 50% practice in clinical settings [14]. The updated curricula included principles of adult teaching and learning focusing on stimulating participatory teaching methodologies – simulation/skill demonstration using low fidelity mannequins, scenarios and role plays. The updated content included respectful maternity care (RMC); emergency obstetrics and newborn care skills; communication using the situation background assessment and recommendation (SBAR) approach to facilitate efficient clinical handover and care; WHO surgical safety checklist to promote theatre safety, teamwork and reduce surgical related deaths; maternal early obstetric warning scores (MEOWS) charts; introduction to maternal and perinatal death surveillance and response (MPDSR). Other updates included students’ assessments and providing effective feedback; academic writing; critical thinking and appraisal; presentation skills; flipped classroom teaching, peer learning and support in teaching (Table 1).

**Table 1:** The updated pre-service midwifery curriculum content

ICM essential competencies for midwifery practice	Updated content
General competencies	Communication using the situation background assessment and recommendation (SBAR); critical thinking and appraisal; assessments and providing effective feedback
Pre-pregnancy and antenatal	Respectful maternity care (RMC); maternal early obstetric warning scores (MEOWS) chart
Care during labor and birth	Emergency obstetrics and newborn care (EmONC) skills; WHO surgical safety checklist
Ongoing care of women and newborns	Emergency obstetrics and newborn care skills; maternal early obstetric warning scores (MEOWS) chart

The overall long-term goal for curricula review is to enable the student to acquire essential competencies for midwifery practise in accordance with ICM core standards. For competent midwifery graduates, midwifery education should be provided within the ICM’s midwifery education standards and enabling environment [14]. Besides, midwifery educators need to possess the updated core competencies to teach theory and practice as recommended by WHO and ICM [14, 15] to be effective so as they can produce graduates with ICM competencies able to practice within the ICM scope for practice [13].

Following the curricula update, a four-day capacity building workshop supported by LSTM for midwifery educators to improve their awareness of the updated curricula and improve their capacity to implement and deliver it was conducted. This was the first ever training specifically for midwifery educators/faculty teaching in university in Kenya on EmONC within the updated curricula. The ICM recommends that the effectiveness and competence of midwifery educators should be reviewed and assessed on a regular basis following an established process [14]. Thus, following the curricula review and update, assessing the capacity of the midwifery educators to implement the competency-based curricula was essential to identify core areas of additional support needed for effective implementation. The objective of the research paper was to assess the change in knowledge, skills and confidence of pre-service university midwifery educators to effectively deliver the updated midwifery curricula after the capacity strengthening workshop in Kenya.

## Methods

### Study design

The study used a quasi-experimental (pre-post) design. Intervention was a four-day training of 30 university midwifery educators in Kenya in EmONC using the adapted 2021 Emergency Obstetric Care and Newborn Care Skilled Health Personnel training package [16]. This package has been used by LSTM in collaboration with Ministries of Health in over 15 LMICs to strengthen the capacity of maternity care providers for quality EmONC [11]. The updated midwifery pre-service curricula are aligned with the 2021 EmONC Skilled Health Personnel training package.

### The updated BScN curricula

The updated curricula were: a 4-year BScN (Direct Entry) and a 2-year BScN upgrading programme (both 2014 versions). The review included updates in the training guidelines (covering teaching content and teaching methods aligned to the intended learning outcomes), assessment tools, student clinical practice training logbooks & exam guidelines. In both curricula, total practical hours were revised and increased from 355 to 570 for the BScN (Direct Entry) and 285 to 320 for the BScN upgrading programme with a reduction in the hours for the theoretical content. Reviews covered all the curricula units in basic nursing sciences, nursing, midwifery and gynaecological nursing, community health, mental health, research, behavioural sciences, leadership and management and curriculum and instruction in nursing among others. The midwifery content was updated to reflect the ICM’s essential competencies for midwifery practice. This content included respectful maternity care, focused antenatal care (FANC) 4 visits to WHO 8 ANC contacts, updates on EmONC signal functions, WHO safe surgical checklist, modified early obstetric warning systems and updates on maternal shock and current recommended management. Other key concepts introduced/updated were: Communication – SBAR (Situation, Background, Assessment & Recommendation), care of mothers after traumatic birth or perinatal loss, postpartum family planning, menstrual hygiene, community midwifery, teenage pregnancy, male partner involvement and maternal and perinatal deaths surveillance and response (MPDSR).

## Intervention

The four-day training was conducted between 31st January – 3rd February 2022 at Moi University School of Nursing and Midwifery, one of the oldest nursing and midwifery training institutions in the country. The training was delivered by a group of eight highly experienced EmONC faculty consisting of EmONC master trainers, course directors and quality assurance officers selected from the ministry of health and midwifery training institutions in Kenya. The training content was delivered through the stimulation participatory teaching methodologies - lectures, role plays, plenary/small group discussions, scenarios and skills drills using various low-fidelity mannequins and equipment. Each small group/skills breakout session had 7–8 participants to allow for sufficient time for hands-on skills training, return demonstrations and skills transfer. Targeted mentorship support sessions were also held for participants who needed some extra guidance/demonstration for certain skills or sessions. At the end of the training, the adapted 2021 Emergency Obstetric Care and Newborn Care Skilled Health Personnel training package electronic facilitator manual was shared with each of the educators as an additional resource to support teaching of EmONC at their institutions. The training and study were approved by the Ministry of Health’s Department of Family Health as part of the strengthening the capacity of pre-service training institutions and monitoring of implementation.

## Study setting

The training intervention was conducted for the university teaching midwifery educators from 27 universities across Kenya. To date, about 28 universities have been approved by the Nursing Council of Kenya to offer the Bachelor of Science in Nursing programme for nurse/midwives training at degree level [17]. Each university has an average of 1–4 educators specialised in midwifery training educated to postgraduate (or ongoing) level even though these staffing levels vary across the institutions.

Midwifery training in Kenya is offered at degree, basic and post basic diploma levels. The Nursing Council of Kenya has approved 121 nursing/midwifery training institutions to train students at degree, basic and post basic diploma level for various branches of nursing & midwifery practice. There are two midwifery degree programs offered at university level in Kenya: Bachelor of Science in Nursing (direct entry and upgrading programs) – an integrated program that trains nurse/midwives; and Bachelor of Science in Midwifery (direct entry and upgrading programs for midwives). The trained nurse/midwives at degree level form part of the skilled health personnel working across the different health care facilities in the country.

## Study participants

The study participants were 30 midwifery educators purposively selected by each university’s midwifery department from all the 27-Bachelor of Science in Nursing training public, faith-based & private universities. One educator was nominated per university due to the limited number of educators per institution, except the host institution that had three educators and one private university that also offers Bachelor of Science in Midwifery that had two educators. The eligibility criteria to participate in the training included midwife lecturers contracted/employed by the universities and actively involved in classroom and clinical teaching of the midwifery modules/units for the Bachelor of Science in Nursing programme for pre-service nursing and midwifery students. Besides classroom teaching, the educators must have been offering clinical supervision and support to students in the clinical settings during clinical placements. This criterion aimed to ensure that the relevant nationally representative subset of nursing and midwifery educators have the capacity to support the role out of the updated midwifery curricula across the country.

## Data collection

### **Informed consent**

was obtained from all the participants about the online pretest and post-test before the start of the training. All participants were assigned unique codes as identifiers during the registration on the training day for participating in the training. All data was anonymous, and this was part of the monitoring of implementation of the pre-service training to strengthen the capacity of midwifery educators and identify areas for additional follow-up support.

Before the start of the training, participants completed the registration process to participate in the training. At this stage, each participant was allocated a unique identity number and colour code (blue, yellow, red or green team) on the identity tag for identification and rotation as a team during skills teaching and demonstration sessions. After completing registration, participants were informed about the anonymous online survey and the benefits of participating in the survey by LSTM’s senior technical officer and training coordinator. It was emphasized that participation in the survey was voluntary, and data collected was strictly confidential. Importantly, data collected was not shared with the institutions’ managers/supervisors neither did it form part of their (educators) performance appraisals.

Upon completing the online survey, participants proceeded to complete the two pretest skills assessments that were set up in the objective structured clinical examinations (OSCE) stations. Each station had a maternal (shoulder dystocia management) and newborn (newborn resuscitation) skill with two assessors per station. The assessors were experienced training faculty and EmONC master trainers trained on conducting assessments and giving feedback. The assessors were paired in each of the four skills stations. Each station had the two skills assessed independently with one assessor per skill. The same online survey and practical skills assessments were repeated immediately after the training on day 4.

## Data collection tools

No participant identifying information was captured on the data collection tools. A unique participant code issued at the registration process was used to facilitate analysis. Three assessments were conducted for knowledge, skills and self-confidence among midwifery educators before and immediately after the training. Knowledge and self-confidence were tested using an online survey tool designed in Google Forms application (supplementary material 1). The online tool was divided into two parts: part A for the knowledge check and part B for the self-confidence check. Online knowledge assessment consisted of 20 multiple choice questions (MCQs) on EmONC and teaching methodologies. These were piloted and validated by a group of 22 lecturers from the Kenya Medical Training College who participated in similar trainings earlier. Questions with item difficulty index (a measure of the proportion of the total examinees who answered an item correctly) of between 0.3 and 0.8 considered good/acceptable were assessed [18].

Part B assessed the self-reported confidence of midwifery educators in teaching pedagogies covering the following six items: lecture, simulation skills teaching and demonstration, scenario, small group discussions, peer teaching and giving effective feedback. A 3-point Likert scale with the alternatives ‘not confident’, ‘somewhat confident’ or ‘extremely confident’ – for rating their confidence in using the six items was provided. The self-rating scale for the perceived confidence of midwifery educators to teach the updated curriculum was adapted from the formerly developed 7-item tool that was used for the evaluation of 51 midwifery and clinical medicine (reproductive health specialty) educators at the mid-level training colleges in Kenya [19]. One component – facilitating online teaching – was excluded in this assessment as the university educators’ training did not have an online/virtual training component. All the six items in the self-confidence survey tool were valid with their obtained Pearson correlation coefficient values greater than the critical value of 0.316 (p < 0.001) at 95% confidence interval. The internal consistency of the self-confidence survey tool, a measure of reliability of the survey tool, was good and acceptable with a Cronbach’s alpha of 0.856 for the surveyed six items (tested using the scores of the six items responded by the 30 midwifery educators during the pre-test assessments before the start of the training).

The two practical skills – one maternal (shoulder dystocia management) and newborn (newborn resuscitation) were performed by all participants and were assessed through objective structured clinical examinations (OSCEs) using online designed checklists. The newborn resuscitation checklist (supplementary material 2) assessed 19 items while the management of shoulder dystocia checklist (supplementary material 3) assessed 14 items, with both checklists adapted from the EmONC skills training OSCEs.

Besides the knowledge, self-confidence and skills assessments, participants also completed an anonymous online post-training assessment immediately after the training on (1) What they intended to change in their practice (or teaching skills) because of the four-day interactive training (2) Make comments/suggestions/key recommendations for improvement of the training. This was for internal program monitoring and evaluation for improving the planning and quality of future capacity strengthening interventions for midwifery educators. All surveys were anonymous.

All the online assessment forms and checklists were designed in Google Forms software. To ensure participant anonymity, no identifying information – name, email address, IP address, and gender were collected on the online forms. Additionally, no signing in was required to access the form.

## Data management and analysis

Data collected from the online google forms were extracted in Microsoft Excel format, cleaned, and exported to SPSS version 26 for statistical analysis. Descriptive statistics were used to describe the findings and are presented in tables.

Online knowledge and skills assessment scores were converted into percentages. As there were two skills (newborn resuscitation and management of shoulder dystocia), a new variable for the average skills scores was calculated by taking the sum of the two skills and dividing by two. Self-reported confidence was rated using a 3-point Likert scale (not confident = 0, somewhat confident = 1 or extremely confident = 2) for teaching pedagogies covering lecture, simulation skills teaching and demonstration, scenario, small group discussions, peer teaching and giving effective feedback. Overall self-reported confidence was dichotomised due to the small cell sizes in some responses for interpretation: the ‘not confident’ and ‘somewhat confident’ groups were categorised as ‘not confident’ (as there were few ‘not confident’ responses) while the ‘extremely confident’ was categorised as ‘confident.’ Shapiro-Wilk test was used to test the normality of data (p > 0.05) as is more appropriate for small sample sizes (< 50 samples). This was to determine the parametric test (paired t-test) or the equivalent non-parametric test (Wilcoxon signed-rank test) to be used to compare the differences in knowledge and skills performance mean scores for the educators before and after the training. The pretest and post-test scores and ratings for each participant were matched. The before mean scores in knowledge and skills were normally distributed (p > 0.05) whereas the after-mean scores were not normally distributed (p < 0.0001). Therefore, the non-parametric Wilcoxon signed-rank test was used to compare the before-after mean scores from the same participants as it does not assume normality in the data [20]. McNemar test was used to determine differences in the proportion of educators’ self-reported confidence (confident or not confident) in applying the different teaching pedagogies [21]. P-values < 0.05 were considered statistically significant at 95% confidence interval.

## Results

### Participant characteristics

A total of 30 educators participated in the capacity building workshop with majority being females (23, 77%). Majority of the educators (23, 77%) had actively participated in classroom teaching less than a month prior to the training. Only 11 (37%) of the educators had participated in a previous hands-on basic EmONC training beforehand with almost all the educators (10, 91%) who had participated in the training having had it over two years ago (Table 2).

**Table 2:** Participant baseline characteristics

Characteristic		Frequency (n = 30)	Percent (100%)
Gender			
	Male	7	23
	Female	23	77
When last classroom teaching done			
	Less than 1 month ago	23	77
	Between 1–3 months	1	3
	Between 3–6 months	3	10
	Over 6 months ago	3	10
Attended previous basic EmONC training			
	Yes	11	37
	No	19	63
When previous EmONC training was attended (n = 11)			
	Less than 6 months ago	1	9
	Between 6–12 months	0	0
	Between 12–24 months	0	0
	Over 24 months	10	91

### Change in knowledge and skills scores

A Wilcoxon signed-rank test showed that there was a statistically significant improvement in the midwifery educators’ performance in the mean knowledge scores from 60.3% (SD ± 13.3) before the training to 88.3% (SD ± 12.3) immediately after the training (P < 0.0001).

On assessment of practical skill, the Wilcoxon signed-rank test showed that there were statistically significant improvements in the performance of newborn resuscitation from 37.9% (SD ± 16.3) to 89.1% (SD ± 11.2), shoulder dystocia from 51.4% (SD ± 25.1) to 88.3% (SD ± 13.8), and the overall skills score from 44.7% (SD ± 16.8) to 88.7% (SD ± 11.3) immediately after the training, p < 0.0001 (Table 3).

**Table 3:** Comparison of midwifery educators’ before and after knowledge and skills assessment mean scores using Wilcoxon signed-rank test

Test	Before		After			
	Mean (%)	SD	Mean (%)	SD	Z-statistic	P-value
Knowledge assessment	60.3	13.3	88.3	12.3	-4.712	< 0.0001*
Newborn resuscitation skill	37.9	16.3	89.1	11.2	-4.796	< 0.0001*
Shoulder dystocia skill	51.4	25.1	88.3	13.8	-4.79	< 0.0001*
Overall skill score	44.7	16.8	88.7	11.3	-4.783	< 0.0001*

SD – standard deviation; * p-value < 0.05 statistically significant

### Change in confidence in using different teaching pedagogies

Midwifery educators self-reported an improvement in their confidence to teach using a variety of the stimulating participatory teaching techniques for both lectures and skills. An exact McNemar’s test determined that there was a statistically significant improvement in the proportion of midwifery educators who were confident immediately after the training in using simulation/demonstration for skill teaching from 11 (36.7%) to 21 (70%), p = 0.006; scenario teaching from 16 (53.3%) to 24 (80%), p = 0.039 and peer teaching and support from 10 (33.3% to 19 (63.3%), p = 0.022. There were also improvements in proportion of midwifery educators who were confident in using the lecture method from 80 − 90% (p = 0.289), facilitating small group discussions from 73.3 to 86.7% (p = 0.344) and giving effective feedback from 60 − 80% (p = 0.146) even though these changes were not statistically significant different from before the training intervention (Table 4).

**Table 4:** Comparison of proportion of confident midwifery educators in using different teaching pedagogies using McNemar’s test

Teaching pedagogy	Before (N = 30)	After (N = 30)	P-value
	Number (Proportion)	Number (Proportion)	
Lecture teaching	24 (80%)	27 (90%)	0.289
Simulation/demonstration	11 (36.7%)	21 (70%)	0.006*
Scenario teaching	16 (53.3%)	24 (80%)	0.039*
Small group discussion	22 (73.3%)	26 (86.7%)	0.344
Peer teaching and support	10 (33.3%)	19 (63.3%)	0.022*
Giving effective feedback	18 (60%)	24 (80%)	0.146

*p < 0.05 statistically significant; N – total number of midwifery educators

## Discussion

Our study findings show that the university midwifery educators’ performance in knowledge and EmONC skills was low before the capacity strenghtening intervention based on the updated curicula but significantly improved after the training. Evidence suggests that skills decline faster within 6–12 months after training [22–25]. Given that majority of the educators had either not had a basic EmONC training before or had it over two years prior to the training, educators need structures, systems and regular continuous professional development opportunities for updating their knowledge and skills through short hands-on clinical refresher trainings and simulation based education to enhance their knowledge, skills and capacity to teach. Large gaps in educator skills have been identified not only in EmONC but also, including basic postnatal care of women and newborns, and the provision of family planning [5]. Thus, such trainings conducted regularly (every 12 months), complemented by mentoring and midwifery educator specific continuous professional development (CPD) programmes that address the full scope of midwifery practice across the continuum of care will be essential to remain relevant in their teaching careers and to ensure quality and competent midwifery graduates [23, 26].

The confidence of educators in using the skills teaching methodologies (simulations and scenarios) was also initially low but greatly improved with the training. As a competency-based program, learning is an active experience and integrating simulation and scenario teaching methods in the curriculum enables midwifery graduates learn how things operate in the real clinical world. These methods promote teaching effectiveness and stimulates student attention and participation, which positively impacts student outcomes [27–29]. Importantly, learning opportunities on patients can be limited, due to the intrusive nature of pregnant women’s health examination and support during birth. Utilisation of simulation-based education can improve students’ confidence, competence and facilitate learning hands-on clinical examination and procedural skills, using realistic part-task and high-fidelity simulators prior to approaching patients – women in labor and childbirth [30–32]. Higher numbers of students in many LMICs and lack of access to clinical training sites, training materials and resources, and including a lack of/low skilled educators hinder the application of the simulation based and participatory teaching methods. Peer teaching and support could well be utilised in training especially where the disparities in student numbers vs. teacher ratios are disproportionately huge. This has the potential to improving students’ critical thinking, learning autonomy, motivation, collaborative and communicative skills [33].

To deliver the updated competency-based curriculum, midwifery educators require more up-to-date hands-on trainings, exposure to equipped skills laboratories to enhance consolidation of theoretical content in classroom and transition to practical and clinical skills practice for students. This has the potential to ensure that there is harmony between the classroom content and real clinical practice in the clinical environment in the hospitals for quality skilled health personnel.

It is expected that competent midwifery educators will employ the theoretical and practical teaching skills to transfer knowledge and skills to the students. The practical component using the humanistic models in education and training enables pre-service midwives to practice and strengthen decision-making and respectful maternity care. This educator approach potentially ensures that pre-service midwives learn the evidence basis for EmONC interventions, practice critical life-saving skills; and transfer their knowledge and skills to their clinical sites [34]. This has a long-term effect of creating competent midwives with capacity to provide quality midifery care for improved maternal and newborn health outcomes.

### Strengths and limitations of the study

To the best of our knowledge, this was the first ever training and faculty development (and assessment of the capacity of educators to teach EmONC) for university midwifery educators on EmONC in Kenya. Although over 13,000 midwifery tutors from countries with the highest rates of maternal and newborn mortality and morbidity across five regions (the Arab States, West and Central Africa, East and Southern Africa, Asia and the Pacific, and Latin America and the Caribbean) have been trained on clinical and teaching skills incuding EmONC through the United Nations Population Fund’s (UNFPA) Maternal and Newborn Health Thematic Fund (MHTF), the assessment on the educators’ capacity and effectiveness of the trainings is lacking [35]. Although the sample size was small, it is imperative that over 95% of the universities that offer nursing and midwifery training at bachelor’s degree level and accredited by the NCK participated in the capacity strengthening intervention. Thus, the results are representative of the country’s capacity to deliver the competency-based EmONC enhanced curriculum at university level. LSTM’s training registration data misses out on additional details such as age, education level, experience (years) and previous continous professional development opportunities attended by the midwifery educators even though the additional variables are important determinants and form part of the standards for effective midwifery education workforce [15, 36, 37]. The data collected was primarily for monitoring and implementation support. Future research on large sample sizes should integrate these factors to determine their overall contribution to competencies of midwifery educators.

### Study implications

The study provides an opportunity for training regulators and policy makers to develop systems to support regular updates in knowledge and skills for midwifery educators to ensure they remain relevant, updated in their professional work and resilient to maintain quality training and education for competent skilled health personnel. Midwifery training institutions require infrastructure – including obstetrics and newborn care simulation training equipment to support skills transfer during training for a competent midwifery graduates. These findings have provided a platform for the design, development and evaluation of a contiuous professional development (CPD) program specific for midwifery educators to update and improve their knowledge, skills, confidence and competence in delivery of their teaching and training in Kenya. Evidence shows that nursing and midwifery educators who participated in CPD activities involving midwifery curricula reviews and teaching developed innovations in teaching & learning and increased their exposure to theoretical teaching and clinical practice. This contributed to provision of EmONC services, led to improved student outcomes and contributed to a quality midwifery workforce in low- and middle-income countries [38–40].

## Conclusion

The training improved the midwifery educators knowledge, skills and confidence in delivering EmONC content using stimulating participatory teaching methods to deliver both theory and practical skills within the updated competency-based pre-service curriculum. To ensure that midwifery educators maintain their competence for practice, there is need for local midwifery regulation and standards for effective midwifery education and training. There is an urgent need to cascade the capacity strengthening of educators to reach more midwifery educators/universities through a sustainable approach. A midwifery educator specific clinical mentorship programme and CPD programme, accredited by the Nursing Council can be designed to achieve this. These strategies maybe critical to ensuring high quality of skilled health personnel at graduation in LMICs.

## Electronic supplementary material

Below is the link to the electronic supplementary material.

Supplementary Material 1

Supplementary Material 2

Supplementary Material 3

## Data Availability

The datasets generated and/or analysed during the current study are not publicly available due to the confidentiality of the data but are available from the corresponding author on reasonable request.

## List of abbreviations

BScN: Bachelor of Science Nursing
CPD: Continuous Professional Development
EmONC: Emergency Obstetric and Newborn Care
ICM: International Confederation of Midwives
LMICs: low- and middle-income countries
NCK: Nursing Council of Kenya
RMC: Respectful Maternity Care
SHP: Skilled Health Personnel
SRMNAH: Sexual, Reproductive, Maternal, Newborn and Adolescent Health
UHC: Universal Health Coverage
UNFPA: United Nations Population Fund
WHO: World Health Organisation
