# Supplementary material for: Improving midwifery educators’ capacity to teach emergency obstetrics and newborn care in Kenya universities: a pre-post study
Source: BMC Med Educ. 2022 Oct 31;22:749. doi: 10.1186/s12909-022-03827-4 (PMC9623932; doi:10.1186/s12909-022-03827-4)
Supplement: Supplementary file 1 — Supplementary Material 1 [file 12909_2022_3827_MOESM1_ESM.pdf]

UNIVERSITY LECTURERS KNOWLEDGE PRETEST

Please provide the details below

*Required

1. Participant number *

2. When did you last teach a midwifery class? *

Mark only one oval.

- Less than 1 month ago
- Between 1 and less than 3 months ago
- Between 3 and less than 6 months ago
- Over 6 months ago

3. Have you undergone a 'hands on' EmONC training session where you practiced on obstetric mannequins before? *

Mark only one oval.

- Yes
- No

4. If yes in above, how long ago was the training?

Mark only one oval.

- Less than 6 months
- Between 6 - 12 months
- Between 12 - 24 months ago
- Over 24 months (2 years) ago

SECTION A: MULTIPLE CHOICE QUESTIONS

Instructions: Please select the best response for each of the questions below.

5. During the closure of a lecture presentation, the teacher should do one of the following: *

Mark only one oval.

- a. Presents material in a clear, logical sequence
- b. Clearly states the expected learning outcomes
- c. Returns to learning outcomes of the lecture
- d. Establishes the importance of skill in practice

6. Over medicalised maternal and newborn care refers to routine use of practices during labour and childbirth that *

Mark only one oval.

- a. Prioritize needs of women over skilled health personnel
- b. Familiar and desirable by women
- c. Do not improve health outcomes for mother and baby though may do harm
- d. Encourage technology with proven benefit

7. In SBAR communication, midwives identify the headline for the referral in the:- *

Mark only one oval.

- a. Background
- b. Situation
- c. Recommendation
- d. Assessment

8. The maneuver employed during childbirth in cases of shoulder dystocia and involves moving the mother to an all fours position with the back arched, widening the pelvic outlet is: - *

Mark only one oval.

- a. Woods' screw maneuver
- b. The McRoberts maneuver
- c. Gaskin maneuver
- d. Jacquemier's maneuver

9. During breech delivery, Lovset’s maneouvre is applied to *

Mark only one oval.

- a. Deliver the legs in frank breech
- b. Deliver shoulder when arms are extended
- c. Deliver stuck head when arms are flexed
- d. Deliver the cheek bones

10. During neonatal resuscitation, chest compressions are given if: *

Mark only one oval.

- a. Baby is unresponsive
- b. Heart rate is less than 60 beats/min
- c. Baby aspirated meconium
- d. Respiration rate is less than 30 breaths/min

11. The precaution that a midwife should take to avoid retained placenta is: - *

Mark only one oval.

- a. Discourage premature pushing during first stage
- b. Administer intravenous fluids during childbirth
- c. Administration of oxytocin following childbirth
- d. Administer prophylactic antibiotics during labor

12. In which of the following conditions will a midwife consider performing a vacuum extraction in a term pregnancy with a cephalic presentation? *

Mark only one oval.

- a. Intrauterine fetal death at full cervical dilatation
- b. Severe pre-eclampsia at full cervical dilatation
- c. Fetal distress at 8cm cervical dilatation
- d. Cord prolapse at 5cm cervical dilatation

13. In the 4-staged method of skills teaching, *

Mark only one oval.

- a. Stage 1 involves the facilitator allowing the candidate to demonstrate the skill
- b. Stage 2 has the facilitator demonstrating the skill 'real time'
- c. Stage 3 has the facilitator demonstrating the skill with candidate commentary
- d. Stage 4 allows the facilitator to demonstrate the skill with commentary

14. During the primary survey for a pregnant mother, which one of the following features can be assessed at the circulation level? *

Mark only one oval.

- a. Feel for air movement in and out of nose/mouth
- b. Auscultation of the fetal heart rate
- c. Listen with stethoscope for air entry
- d. Call the patient to check if unresponsive

15. Mrs. X was admitted at the CEOC facility at 9am and diagnosed with features of severe pre-eclampsia and started on the recommended Magnesium Sulphate as per national protocols. She was monitored and had a successful spontaneous vaginal birth at 1pm though she experienced a fit at 4pm while on still on the Magnesium Sulphate treatment. Which of the following is the correct management option for Mrs. X? *

Mark only one oval.

- a. Continue with Magnesium Sulphate maintenance dose and stop at 9am the following day
- b. Continue with Magnesium Sulphate maintenance dose and stop at 1pm the following day
- c. Continue with Magnesium Sulphate maintenance dose and stop at 4pm the following day
- d. Continue with Magnesium Sulphate maintenance dose and stop at 1am the following day

16. The following statement is true regarding obstetric hemorrhages *

Mark only one oval.

- a. In every case of placental abruption, be prepared for postpartum hemorrhage
- b. Conduct digital vaginal examination first to diagnose placenta praevia
- c. If cervix is fully dilated but fetal heart rate is abnormal, do not attempt vaginal delivery
- d. Always commence IV fluids then secure airway and breathing in hemorrhage

17. Which of the following statements is true about newborn resuscitation? *

Mark only one oval.

- a. Stimulate newborn using a dry warm cloth then clear meconium-filled airway
- b. Start chest compressions if newborn is not breathing with open airway immediately
- c. Continue ventilation for 1 minute if heart rate is more than 60 beats/minute
- d. Consider chest compressions if heart rate is more than 60 but less than 100/minute

18. During the primary survey of a critically ill pregnant woman, the following assessments are performed: *

Mark only one oval.

- a. Breathing, urine output and past medical history
- b. Respiratory rate, fetal heart rate and blood culture
- c. Responsiveness of the mother and past surgical history
- d. Blood pressure, fetal heart rate and responsiveness

19. When using maternity early obstetrics warning score chart, which of the following statements is true? *

Mark only one oval.

- a) Repeat observations half hourly with a score of less than 2
- b) Perform routine 4-hourly observations with a score of 3 or more
- c) Call the next senior health provider if the score is less than 3
- d) Half hourly observations to be taken with a score of 2 or 3

20. Which one of the following statements is true when closing a scenario practice? *

Mark only one oval.

- a. Get a learner to do it as they explain what they are doing
- b. Explain the scenario as a co-facilitator demonstrates simultaneously
- c. Ask the group to explain what would have been done better
- d. Observes the attitudes and behaviours of the group

21. Before performing Vacuum Extraction, the midwife should ensure that the following conditions are met: - *

Mark only one oval.

- a) Fetal gestation at 34/40, full dilatation of the cervix and ruptured fetal membranes
- b) Full dilatation of cervix, cephalic presentation and fetal head more than 4/5 palpable abdominally
- c) Fetal gestation at 38/40, full dilatation of the cervix and empty bladder
- d) Face presentation, empty bladder and full dilatation of the cervix

22. Which one of the following statements is true regarding the WHO surgical safety checklist? *

Mark only one oval.

- a) It can easily compromise teamwork and communication during obstetric operations
- b) It is used for cases that require elective preparation in advance and not emergencies
- c) It should be used for every patient, at every step peri-operatively in a hospital
- d) Healthcare workers should memorize it to avoid delays during operations and risks

23. During administration of Magnesium Sulphate to a woman in labor with impending eclampsia, the midwife uses a loading dose of: *

Mark only one oval.

- a) 4g of 20% IV
- b) 1g of 20% IV
- c) 5g of 50% IM
- d) 10g of 20% IV

24. Which one of the following is an indication for giving cardiopulmonary resuscitation in an adult? *

Mark only one oval.

- a. Absence of breathing in the presence of an open airway
- b. Presence of circulation in the absence of breathing
- c. Presence of circulation in the presence of weak chest movements
- d. Patient subconscious in the absence of a defibrillator

SECTION B: SELF-CONFIDENCE IN TEACHING METHODOLOGIES

25. How confident do you feel teaching using the following methods/skills? *

Mark only one oval per row.

	Not confident	Somehow confident	Confident
**Lecture**	⬭	⬭	⬭
**Simulation/role play**	⬭	⬭	⬭
**Scenario**	⬭	⬭	⬭
**Small group discussion**	⬭	⬭	⬭
**Use of peer teaching**	⬭	⬭	⬭
**Give effective feedback**	⬭	⬭	⬭

This content is neither created nor endorsed by Google.

Google Forms
