# Supplementary material for: Improving midwifery educators’ capacity to teach emergency obstetrics and newborn care in Kenya universities: a pre-post study
Source: BMC Med Educ. 2022 Oct 31;22:749. doi: 10.1186/s12909-022-03827-4 (PMC9623932; doi:10.1186/s12909-022-03827-4)
Supplement: Supplementary file 2 — Supplementary Material 2 [file 12909_2022_3827_MOESM2_ESM.pdf]

# OBSTETRIC EMERGENCIES - SHOULDER DYSTOCIA

Scenario

*Required

1. Participant number *

Scenario: Mrs D is 26 years old, G2P1, 41 weeks gestation. She is in active labour with cephalic presentation and slow progress. After full dilatation, the baby's head appears but the shoulders fail to deliver.

2. Show me what you would do.

*Mark only one oval per row.*

| | 0 | 1 |
|---|---|---|
| **Inform the mother and** | ⬭ | ⬭ |
| **Call for help** | ⬭ | ⬭ |

3. Please tell me what you would do next to deliver the baby.

*Mark only one oval per row.*

| | 0 | 1 |
|---|---|---|
| **Place the woman in McRoberts** | ⬭ | ⬭ |

4. Describe McRoberts position.

*Mark only one oval per row.*

| | 0 | 1 |
|---|---|---|
| **Knees to chest** | ⬭ | ⬭ |
| **Abduct rotate legs outwards** | ⬭ | ⬭ |

5. What else would you combine with the McRoberts position?

*Mark only one oval per row.*

| | 0 | 1 |
|---|---|---|
| **Apply suprapubic pressure** | ⬭ | ⬭ |
| **Pressure to the correct side using heel of hand** | ⬭ | ⬭ |

6. What other manouevres could you use?

*Mark only one oval per row.*

| | 0 | 1 |
|---|---|---|
| **Internal rotational manoeuvres - apply pressure to the anterior shoulder in direction of sternum.** | ⬭ | ⬭ |
| **Apply pressure to the posterior shoulder in direction of the sternum** | ⬭ | ⬭ |
| **Deliver the posterior arm - grasp humerous of posterior arm, keep arm flexed, sweep across chest** | ⬭ | ⬭ |

7. If these don't work, tell me what other techniques you could try?

*Mark only one oval per row.*

| | 0 | 1 |
|---|---|---|
| **Turn woman onto 'all fours'** | ⬭ | ⬭ |
| **Fracture clavical** | ⬭ | ⬭ |

8. What complications should you be alert for after delivery?

*Mark only one oval per row.*

| | 0 | 1 |
|---|---|---|
| **Maternal - PPH; tears to birth canal** | ◯ | ◯ |
| **Fetal - Erbs palsy; fetal brain damage; fractured humerus/clavicle)** | ◯ | ◯ |

This content is neither created nor endorsed by Google.

Google Forms
