# Supplementary material for: Improving midwifery educators’ capacity to teach emergency obstetrics and newborn care in Kenya universities: a pre-post study
Source: BMC Med Educ. 2022 Oct 31;22:749. doi: 10.1186/s12909-022-03827-4 (PMC9623932; doi:10.1186/s12909-022-03827-4)
Supplement: Supplementary file 3 — Supplementary Material 3 [file 12909_2022_3827_MOESM3_ESM.pdf]

NEWBORN RESUSCITATION POST TEST

*Required

1. Participant number *

Scenario: A woman, referred to your facility, has been in labour for 17 hours. The baby is delivered by C/S (and passed to you). It is floppy, unresponsive and white. You have been asked to go to theatre to take the baby.

2. Please demonstrate what you would do.

Mark only one oval per row.

	0	1
**Call for help**		
**Check the time**		
**Dry/stimulate**		
**Wrap in a fresh towel**		
**Cover head with newborn hat**		

3. You reassess and find that the baby is still unresponsive, what will you do next?

Mark only one oval per row.

	0	1
**Open airway (ask how)**	◯	◯
**Place baby in a sniffing position**	◯	◯
**Assess heart rate with stethoscope**	◯	◯

4. The baby is not breathing, has HR of 90bpm and is blue. What would you do next?

Mark only one oval per row.

	0	1
**Choose correct size bag and mask**	◯	◯
**Assemble bag and mask**	◯	◯
**Position correctly on the baby's face**	◯	◯
**Give inflation breaths**	◯	◯
**Give 40 - 60 breaths in a minute**	◯	◯

5. You have reassessed the heart rate is now 50bpm and the baby is still not breathing. Please show me what would you do next

Mark only one oval per row.

	0	1
**Do CPR**	◯	◯

6. Demonstrate chest compressions

Mark only one oval per row.

	0	1
**Correct positioning of the hands/thumbs**	◯	◯
**Correct depth (1/3rd depth) and ratio of compressions (1:3)**	◯	◯
**Correct rate (quickly but giving time for cardiac refilling)**	◯	◯

7. When can compressions be stopped?

Mark only one oval per row.

	0	1
**When HR is above 60bpm and rising**	◯	◯
**No gasping/breathing after 20 mins or gasping but noregular breathing after 30 mins**	◯	◯

This content is neither created nor endorsed by Google.

Google Forms
